# Intramembrane proteases protect from atherosclerosis

**DOI:** 10.18632/aging.102342

**Published:** 2019-10-04

**Authors:** Torben Mentrup, Bernd Schröder

**Affiliations:** 1 Institute for Physiological Chemistry, Technische Universität Dresden, D-01307 Dresden, Germany

**Keywords:** atherosclerosis, LOX-1, intramembrane proteolysis, signal peptide peptidase-like 2 proteases, endothelial activation

Cardiovascular diseases with their clinical manifestations myocardial infarction, ischemic stroke and peripheral arterial disease still represent a leading cause of death [1]. Pathophysiological hallmark of these conditions are atherosclerotic plaques, which progressively develop in the vascular wall. They are characterized by lipid deposition, macrophage infiltration and arterial wall thickening [2] and finally lead to occlusion of the vascular lumen and induce ischemic insults.

The activation of endothelial cells represents one of the first events in this pathophysiological sequence, which initiates adhesion and recruitment of immune cells [2]. Oxidized LDL particles can activate endothelial cells, which is largely mediated by the Lectin-like oxidized low density lipoprotein receptor 1 (LOX-1). LOX-1 is a single-spanning transmembrane protein with type II-orientation (N-terminus facing the cytosol) (Figure 1), which belongs to the family of C-type lectin receptors [3]. Upon binding of oxLDL, LOX-1 triggers several intracellular signaling pathways including MAP kinases. In endothelial cells, this induces the upregulation of adhesion molecules facilitating leukocyte recruitment and thereby the initiation of plaque development. Beyond that, LOX-1 is also expressed in other cell types including smooth muscle cells and macrophages where it triggers a pro-atherogenic and pro-fibrotic transcriptional program. In LOX-1 deficient mice, atherosclerotic plaque development is diminished [4], whereas it is enhanced in LOX-1 overexpressing mice [5], thus providing unambiguous evidence for the pathophysiological relevance of this receptor.

**Figure 1:**
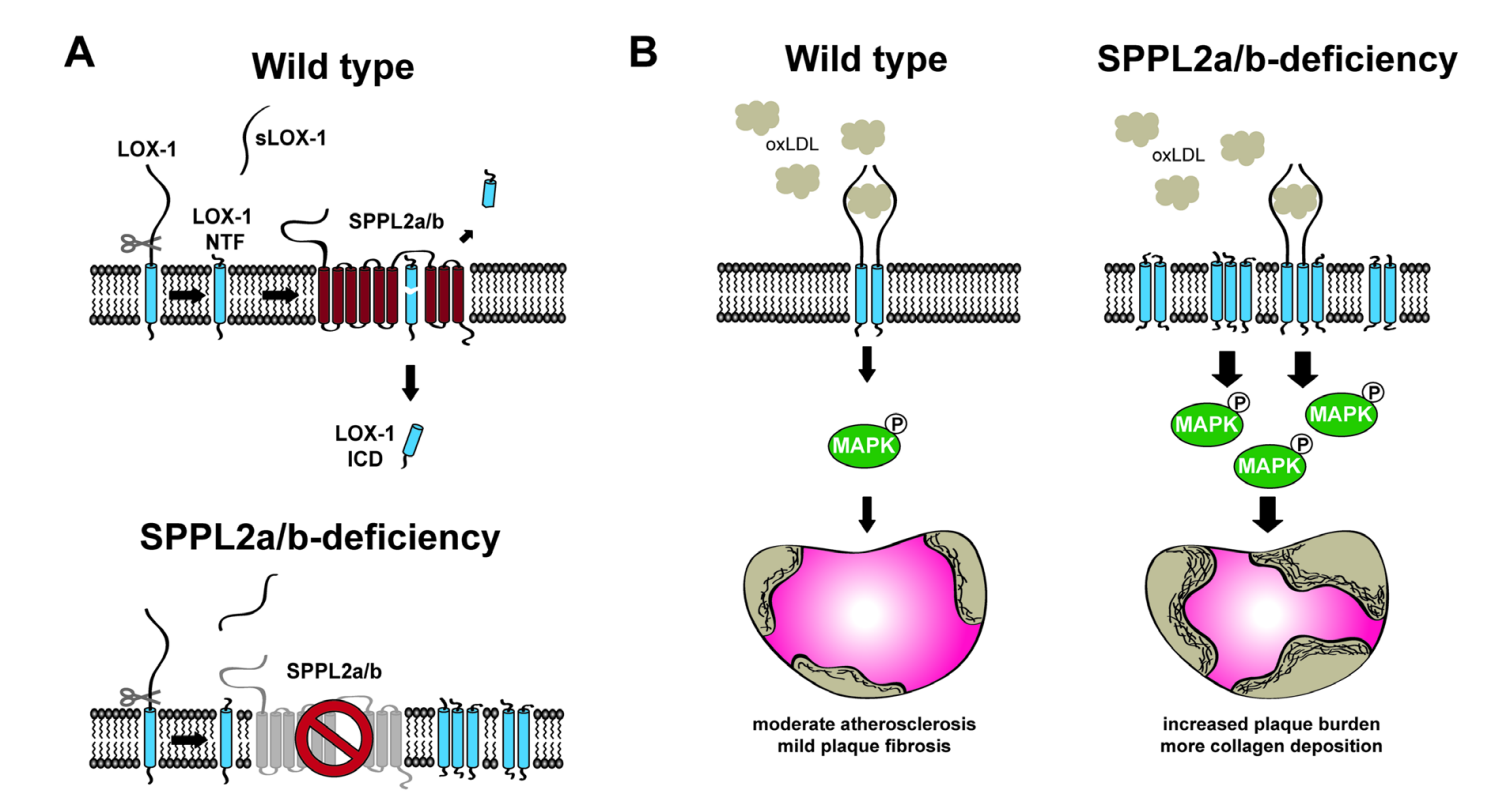
**LOX-1 cleavage by the intramembrane proteases SPPL2a/b is athero-protective.** (**A**) After proteolytic cleavage of the LOX-1 ectodomain, the remaining N-terminal fragment (NTF) is further processed by the intramembrane proteases Signal peptide peptidase-like 2a/b (SPPL2a/b). Thereby, the LOX-1 intracellular domain (ICD) is released into the cytosol. In SPPL2a/b-deficient cells, the uncleaved LOX-1 NTF accumulates in the membrane. (**B**) LOX-1 is a receptor for oxidized LDL (oxLDL). Following binding of oxLDL, LOX-1 triggers pro-atherogenic MAP kinase signalling, which can be enhanced by accumulating NTFs in SPPL2a/b-deficient cells. In parallel, the LOX-1 NTF can signal autonomously in a ligand-independent way.

Despite the critical importance of LOX-1, the molecular mechanisms how this receptor induces signal transduction and how this process is regulated remain incompletely understood. With regard to the latter, we recently identified proteolytic processing as a major regulatory mechanism [6]. We could show that processing of the LOX-1 ectodomain by different proteases leads to the continuous generation of membrane-bound N-terminal fragments (NTFs) from this receptor (Figure 1A). For further degradation and clearance from the membrane, these LOX-1 NTFs depend on the intramembrane proteases Signal peptide peptidase-like 2a and –b (SPPL2a/b). These enzymes belong to the SPP/SPPL family of intramembrane proteases and are capable of cleaving substrate proteins within their transmembrane segments [7]. Thereby, cleavage fragments are released to either side of the membrane. Consequently, in the absence of SPPL2a/b the uncleaved LOX-1 NTFs accumulate in the membrane, which is prominently observed in aortae of SPPL2a/b-deficient (dKo) mice (Figure 1A) [6].

With regard to the function of LOX-1, blocking the intramembrane cleavage of the LOX-1 NTF enhances oxLDL-induced signalling in endothelial cells (Figure 1B). This effect correlates with a molecular interaction between the accumulating LOX-1 NTF and the full length LOX-1 protein and a more efficient signalling activation by these complexes. Importantly, we observed that the LOX-1 NTF is able to activate MAP kinases also independently of the LOX-1 full-length protein. This autonomous signaling activity of the LOX-1 NTF is critically associated with its ability to dimerize and/or oligomerize, which depends on determinants within the transmembrane segment. At the downstream level, the NTF-induced MAP kinase activation enhances expression of several target genes including adhesion molecules, chemokines and growth factors altogether promoting a pro-atherogenic state. These findings have revealed a previously unrecognized mode of LOX-1 signaling, which contributes to the overall pro-atherogenic and pro-fibrotic function of the receptor. The NTF, which is generated in the course of receptor degradation, is well detectable in the aortae of wild type mice and its abundance increases under atherogenic conditions. Since the LOX-1 NTF signals constitutively without the need of a ligand, its proteolytic control by the intramembrane proteases SPPL2a/b is of critical importance.

In agreement, SPPL2a/b dKo developed larger plaques than control mice following atherosclerosis induction with a high cholesterol Western type diet in combination with PCSK9 overexpression [6]. In addition, plaques were more fibrotic and exhibited a higher collagen content. Biochemical analysis revealed enhanced MAP kinase activation and increased expression of the adhesion molecule ICAM-1 in plaques from the dKo mice, which corresponds to the effects induced by the LOX-1 NTF at the cellular level. These findings unequivocally confirm in an *in vivo* context that the proteases SPPL2a/b are required to control and limit the pro-atherogenic effects of LOX-1 and by this means the development of atherosclerosis. Since also human LOX-1 is proteolyzed by SPPL2a/b, the described pathways unravelled in mice are likely relevant also in humans. The spectrum of substrates and pathophysiological functions of these enzymes is still emerging. LOX-1 represents the first *in vivo* validated substrate of SPPL2b. In contrast, a prominent role of SPPL2a in immune cells by processing NTFs of CD74, which facilitates assembly and trafficking of MHCII complexes, has been described earlier [8] identifying this protease as a potential therapeutic target to treat autoimmunity. At least in mice both SPPL2a and SPPL2b contribute to LOX-1 cleavage suggesting that even upon therapeutic SPPL2a inhibition the impact on LOX-1 NTF turnover and pro-atherogenic signalling may remain in a tolerable range as long as SPPL2b activity is spared. With regard to the prevention of atherosclerosis, any way to enhance the SPPL2a/b-mediated turnover of the LOX-1 NTF can be expected to be athero-protective and may thus be beneficial. Currently, the regulation of SPP/SPPL intramembrane proteases is poorly understood. Further work will be needed to elucidate how these signaling-modulating enzymes are regulated themselves and if these pathways can be therapeutically exploited.

## References

[1] Herrington W, et al. Circ Res. 2016; 118:535–46. 10.1161/CIRCRESAHA.115.307611 26892956

[2] Gimbrone MA Jr, et al. Circ Res. 2016; 118:620–36. 10.1161/CIRCRESAHA.115.306301 26892962 PMC4762052

[3] Xu S, et al. Cell Mol Life Sci. 2013; 70:2859–72. 10.1007/s00018-012-1194-z 23124189 PMC4142049

[4] Mehta JL, et al. Circ Res. 2007; 100:1634–42. 10.1161/CIRCRESAHA.107.149724 17478727

[5] Akhmedov A, et al. Eur Heart J. 2014; 35:2839–48. 10.1093/eurheartj/eht532 24419805

[6] Mentrup T, et al. J Exp Med. 2019; 216:807–30. 10.1084/jem.20171438 30819724 PMC6446863

[7] Mentrup T, et al. Biochim Biophys Acta Mol Cell Res. 2017; 1864:2169–82. 10.1016/j.bbamcr.2017.06.007 28624439

[8] Schneppenheim J, et al. J Exp Med. 2013; 210:41–58. 10.1084/jem.20121069 23267015 PMC3549707

